# Differential impact of digital therapy on storage and voiding LUTS: A post‐hoc IPSS analysis from the BEST randomized controlled trial

**DOI:** 10.1002/bco2.70069

**Published:** 2025-09-25

**Authors:** Sandra Schönburg, Christian Gratzke, Kurt Miller, Erik Krieger, Patrick Papp, Laura Wiemer

**Affiliations:** ^1^ BG Klinikum Bergmannstrost Halle (Saale) Germany; ^2^ Department of Urology and Kidney Transplantation Martin Luther University Halle (Saale) Germany; ^3^ Department of Urology University of Freiburg Germany; ^4^ Charité, Department of Urology University of Berlin Germany; ^5^ Kranus Health GmbH Munich Germany

**Keywords:** BPH, Digital therapy, IPSS, male LUTS, OAB

## Abstract

**Objective:**

To investigate whether storage or voiding symptoms respond more favourably to the use of Kranus Lutera, the first app‐based digital therapeutic for male lower urinary tract symptoms (LUTS), using detailed item‐level analysis of the IPSS questionnaire.

**Materials and Methods:**

The present data represent a post‐hoc analysis of the results of the BEST trial, a randomized controlled study evaluating the efficiency of the digital therapy Kranus Lutera. The study period lasted 12 weeks, conducted between 04/2023 and 11/2023. We assessed the mean change from baseline to 12 weeks for each of the seven IPSS items. Voiding symptoms (items 1, 3, 5 and 6) and storage symptoms (items 2, 4 and 7) were analysed separately.

**Results:**

Participants using the digital therapeutic demonstrated statistically significant improvements across all IPSS items. Compared to the control group, the intervention group showed a significant and clinically relevant improvement in the primary endpoint (IPSS), with an overall reduction of −7.0 points (95% CI: −8.1 to −5.9, p < 0.0001). Notably, improvements in storage symptoms were consistently larger than those in voiding symptoms. The analysis of individual IPSS questions showed the greatest changes in the overall cohort for questions 1, 2 and 7 (each p < 0.0001). Patients with the single diagnosis BPH (N40) showed the greatest score reduction in questions 2 and 5 (each p < 0.0001), patients with OAB (N32.8) in questions 2, 4 and 7 (each p < 0.0001) and patients with BPH and OAB (N40 + N32.8) in questions 2, 3 and 7 (question 2 and 3 p < 0.0001, question 7 p = 0.0015). According to the analysis of individual IPSS questions, the greatest improvements were observed in frequency, nocturia and the feeling of incomplete bladder emptying.

**Conclusion:**

These findings suggest that a structured app‐based therapeutic may exert a stronger effect on storage symptoms than voiding symptoms in men with LUTS. This study confirms the value of the digital therapy as an integral part of the standard care for patients with male LUTS.

## 1 INTRODUCTION

The International Prostate Symptom Score (IPSS) is a widely used questionnaire for evaluating lower urinary tract symptoms (LUTS) and prostate‐related voiding dysfunction in men.^1^ It includes questions on bladder emptying as well as storage symptoms. Specifically, it assesses the quality of bladder emptying (1), frequency (pollakiuria) (2), voiding interruption (3), urgency (4), the strength of the urinary stream (5), potential initiation difficulties (6) and nocturia (7). Storage symptoms, particularly frequency, urgency and nocturia, are commonly identified as the most bothersome by patients and significantly reduce their quality of life. These symptoms are captured by questions 2, 4 and 7. The IPSS is applied in clinical practice as well as in research studies, particularly in approval studies.

Kranus Lutera is the first Digital Health Application (dt. Digitale Gesundheitsanwendung, DiGA) for the treatment of male LUTS. The core of this app‐based therapy is a behaviour modification program. It includes educational content, various techniques to suppress urgency, a bladder diary, relaxation techniques and pelvic floor exercises.^2^ The BEST‐study, which served as the approval study for this health application, demonstrated a significant benefit of the intervention group compared to the control group.^3^ The study included patients with benign prostate hyperplasia (BPH) and/or overactive bladder (OAB). However, it remains unclear which specific symptoms are most responsive to digital therapy. This post‐hoc analysis aims to evaluate this question.

## 2 MATERIAL AND METHODS

The BEST‐study was a two‐arm, randomized, controlled, bicentric, single‐blinded study assessing Kranus Lutera as treatment for male LUTS. The study period lasted 12 weeks, and patient enrolment occurred between April and November 2023. Inclusion and exclusion criteria as well as the relevant patient demographics are presented in Table 1. The patient demographics between the two groups were balanced. The full analysis set has already been published.^3^ Participants in the intervention group (IG) received standard of care plus access to the digital therapy Kranus Lutera, while participants in the control group (CG) received standard of care and were granted access to the digital therapy after the primary data collection period of 12 weeks. A sham app was not used. The primary endpoint was assessed as the improvement according to the validated IPSS questionnaire. Secondary endpoints included changes in the OAB‐q SF part 1 (for LUTS symptoms) and OAB‐q SF part 2 (for health‐related quality of life). Treatment failure was defined as an increase in the IPSS score by ≥3 points and/or an increase in OAB‐q SF part 1 by ≥3.3 points (11% OAB), acute urinary retention or the initiation of a new LUTS‐specific medication or surgery.

**TABLE 1:** Inclusion and exclusion criteria as well as patient demographics of the BEST‐study.

Inclusion criteria	Exclusion criteria
Men with LUTS, OAB‐q‐SF Part 1 ≥ 18 or IPSS ≥ 4	Recurrent urinary retention
Age ≥ 18 years	Recurrent urinary tract infections
Proficiency in the German language	Bladder stones
Internet access	Conservatively uncontrollable, recurrent macroscopic haematuria
Tablet or smartphone access	Dilation of the upper urinary tract, impaired kidney function or kidney failure due to obstructive bladder emptying disorder
Informed consent	Newly initiated medication therapy for voiding symptoms (α‐blockers, 5‐α reductase inhibitors, anticholinergics, beta‐3 receptor agonists, phytopharmaceuticals) in the last 4 weeks
	Inability to physically participate in the program
	Patients who are unable to understand and independently sign the informed consent

Relevant patient demographics of the overall cohort*			
Characteristic	Intervention group (n = 112)	Control group (n = 125)	Total (n = 237)
Age ≤65 years ‐ no. (%)	80 (71.4)	86 (68.8)	166 (70.0)
Age >65 years ‐ no. (%)	32 (28.6)	39 (31.2)	71 (30.0)
Current medication for male LUTS ‐ no. (%)	31 (27.7)	38 (30.4)	69 (29.1)
Previous therapies for male LUTS ‐ no. (%)	23 (20.5)	27 (21.6)	50 (21.1)

* Relevant demographics for the post hoc analysis; the full analysis set has already been published.^3^

The IPSS is the most widely used international questionnaire for quantifying the severity of male LUTS and prostate‐related voiding dysfunction. The IPSS consists of seven questions related to urination, each rated from 0 (“never”) to 5 (“almost always”). It includes questions on voiding (question 1, 3, 5 and 6) as well as storage symptoms (question 2, 4 and 7), typically referring to the past month. The maximum possible score is 35 points, with higher values indicating more severe symptoms.^1^ The OAB‐q questionnaire and its short form (OAB‐q SF) specifically focus on OAB symptoms.^4^, ^5^, ^6^ The OAB‐q SF consists of six questions assessing symptom bother, which includes voiding symptoms, incontinence and nocturia, with higher scores indicating more severe symptoms. Additionally, the questionnaire includes 13 questions evaluating health‐related quality of life, with higher transformed scores indicating better quality of life.

The study was reviewed and positively evaluated by the Ethics Committee of the Medical Faculty of Martin Luther University Halle‐Wittenberg (Ethics Committee Approval No.: 2022–139) and the participating Ethics Committee of Albert Ludwig University Freiburg (Application No. EK‐Freiburg: 23–1219‐S1‐AV) and was registered in the German Clinical Trials Register (DRKS: DRKS00030935).

The present investigation is a post‐hoc analysis of the BEST‐study. The objective was to evaluate which symptoms, according to the individual IPSS questions, primarily respond to the digital therapy. All underlying statistical analyses were conducted using SAS® software (Version 9.4). Statistical significance was set at 0.05 (two‐sided). For the three confirmatory tested endpoints, the change from baseline to study end (CfB) was determined as “post‐baseline” and analysed using covariance analysis (ANCOVA). Additionally, the corresponding 95% confidence intervals were calculated.

## 3 RESULTS

For the overall population, the intervention group showed a significant improvement in the IPSS score from 17.44 to 10.52 points during the treatment period compared to the control group, corresponding to a reduction of 6.92 score points. Since the CG remained almost unchanged at −0.02 points (Baseline: 17.70 points; 12 weeks after therapy: 17.67 points), the LS Mean Difference was −7.0 points, demonstrating a clear superiority of the intervention. This result was statistically significant (Table 2). Regarding symptom burden according to OAB‐q SF symptom bother, the IG improved from 49.94 to 30.15 points, representing a symptom reduction of −18.6 points. Again, the CG remained nearly unchanged, showing only minimal variation (51.60 to 49.91 points). The LS Mean Difference for OAB‐q SF part 1 was −18.6 points, which was statistically significant (Table 2). For health‐related quality of life according to OAB‐q SF part 2, a similar result was observed. The IG showed a substantial improvement from 61.26 to 79.26 points (+17.2 points), whereas the CG remained relatively stable (59.00 to 60.46 points). Consequently, the LS Mean Difference of +17.2 points favoured the IG, indicating a significant improvement in quality of life for the IG compared to the CG (Table 2). The digital therapy led to a significant improvement in LUTS according to IPSS over 12 weeks, a substantial reduction in symptom burden according to OAB‐q SF part 1, and an improved quality of life according to OAB‐q SF part 2.

**TABLE 2:** Changes of the IPSS score, the OAB‐q SF part 1 and part 2 score for the overall cohort, IG: n = 112 pts., CG: n = 125 pts.

		Intervention group (n = 112)		Control group (n = 125)	
		Baseline	Week 12	Baseline	Week 12
**Absolute Change of the IPSS score from baseline to week 12 (point)**	Mean	17.44	10.52	17.70	17.67
	±SD	6.16	4.96	6.02	6.43
	LS Mean Difference	**−7.0**			
	95% CI	−8.1; −5.9			
	p value (ANCOVA)	<0.0001			
**Absolute Change of the OAB‐q SF score part 1 from baseline to week 12** **(points)**	Mean	49.94	30.15	51.60	49.91
	±SD	19.72	19.35	18.85	19.24
	LS Mean Difference	**−18.6**			
	95% CI	−22.2; −15.0			
	p value (ANCOVA)	<0.0001			
**Absolute Change of the OAB‐q SF score part 2 from baseline to week 12** **(points)**	Mean	61.26	79.26	59.00	60.46
	±SD	18.55	15.49	17.12	17.47
	LS Mean Difference	**+17.2**			
	95% CI	14.18; 20.16			
	p value (ANCOVA)	<0.0001			

The IG demonstrated a greater improvement in IPSS questions than the CG across the total cohort and all subgroups (BPH + OAB, OAB, BPH), as evidenced by the negative LS Mean Differences, which were statistically significant. A closer examination of the individual IPSS questions revealed improvements in specific symptoms. As shown in Table 3, significant improvements were observed for frequency (LS Mean Difference [95% CI]: −1.2 [−1.5; −1.0]), nocturia (LS Mean Difference [95% CI]: −1.1 [−1.4; −0.8]) and the sensation of incomplete bladder emptying (LS Mean Difference [95% CI]: −1.0 [−1.2; −0.7]).

**TABLE 3:** IPSS single question evaluation for the overall cohort, the BPH + OAB group, the BPH group and the OAB group.

IPSS‐question	Meaning	LS Mean Difference (95% KI)	p value
**Overall cohort – IG: n = 112 pts., CG: n = 125 pts**.			
1	Incomplete bladder emptying	−1,0 (−1,2; −0,7)	p < 0,0001
2	Frequency (Pollakiuria)	−1,2 (−1,5; −1,0)	p < 0,0001
3	Intermittency	−0,9 (−1,2; −0,7)	p < 0,0001
4	Urgency	−0,9 (−1,1; −0,6)	p < 0,0001
5	Weak Stream	−0,9 (−1,2; −0,7)	p < 0,0001
6	Straining	−0,8 (−1,1; −0,6)	p < 0,0001
7	Nocturia	−1,1 (−1,4; −0,8)	p < 0,0001
**Group BPH+OAB=N40 + N32.8 – IG: n = 26 pts., CG: n = 27 pts**.			
1	Incomplete bladder emptying	−1,0 (−1,7; −0,3)	p = 0,0036
2	Frequency (Pollakiuria)	−1,3 (−1,8; −0,8)	p < 0,0001
3	Intermittency	−1,3 (−1,9; −0,7)	p < 0,0001
4	Urgency	−1,0 (−1,6; −0,4)	p = 0,0022
5	Weak Stream	−0,8 (−1,3; −0,3)	p = 0,0029
6	Straining	−0,9 (−1,2; −0,6)	p < 0,0001
7	Nocturia	−1,2 (−1,9; −0,5)	p = 0,0015
**Group BPH=N40 – IG: n = 54 pts., CG: n = 56 pts**.			
1	Incomplete bladder emptying	−0,9 (−1,3; −0,6)	p < 0,0001
2	Frequency (Pollakiuria)	−1,1 (−1,4; −0,8)	p < 0,0001
3	Intermittency	−0,9 (−1,2; −0,5)	p < 0,0001
4	Urgency	−0,6 (−1,0; −0,3)	p = 0,0011
5	Weak Stream	−1,0 (−1,4; −0,6)	p < 0,0001
6	Straining	−0,9 (−1,2; −0,6)	p < 0,0001
7	Nocturia	−0,9 (−1,4; −0,5)	p = 0,0001
**Group OAB=N32.8 – IG: n = 32 pts., CG: n = 42 pts**.			
1	Incomplete bladder emptying	−1,1 (−1,5; −0,6)	p < 0,0001
2	Frequency (Pollakiuria)	−1,3 (−1,8; −0,9)	p < 0,0001
3	Intermittency	−0,8 (−1,3; −0,3)	p = 0,0011
4	Urgency	−1,3 (−1,7; −0,8)	p < 0,0001
5	Weak Stream	−0,8 (−1,3; −0,4)	p = 0,0003
6	Straining	−0,8 (−1,2; −0,3)	p = 0,0010
7	Nocturia	−1,3 (−1,8; −0,8)	p < 0,0001

The greatest improvement was found for frequency (IPSS Question 2), with LS Mean Differences of −1.3 in the BPH + OAB and OAB subgroup (Table 3). Nocturia (IPSS Question 7) also showed consistently high improvements, with LS Mean Difference values of −1.2 and −1.3 in the BPH + OAB and OAB subgroup. A consistent improvement was also observed for the sensation of incomplete bladder emptying (IPSS Question 1), with LS Mean Differences of −1.0 and −1.1 in the BPH + OAB and OAB subgroup, respectively. Other symptoms, such as interrupted stream (Question 3), urgency (Question 4), weak stream (Question 5) and hesitancy (Question 6), also responded well to the digital therapy, as demonstrated by their respective negative LS Mean Differences, which were all statistically significant, although the improvements were not as pronounced as for the aforementioned symptoms.

When comparing the subgroups of overactive bladder (OAB, N32.8, Table 3) and benign prostatic hyperplasia (BPH, N40, Table 3), a stronger effect was observed in patients with OAB, particularly in urgency (LS Mean Difference [95% CI]: −1.3 [−1.7; −0.8]) and nocturia (LS Mean Difference [95% CI]: −1.3 [−1.8; −0.8]). Patients with single BPH without storage symptoms experienced a slightly lower improvement in urgency (LS Mean Difference [95% CI]: −0.6 [−1.0; −0.3]), while other values were comparable. All reported improvements were statistically significant (p < 0.0001). Only a few p‐values were slightly higher but still statistically significant, such as urgency in the BPH group with p = 0.0011.

**FIGURE 1:**
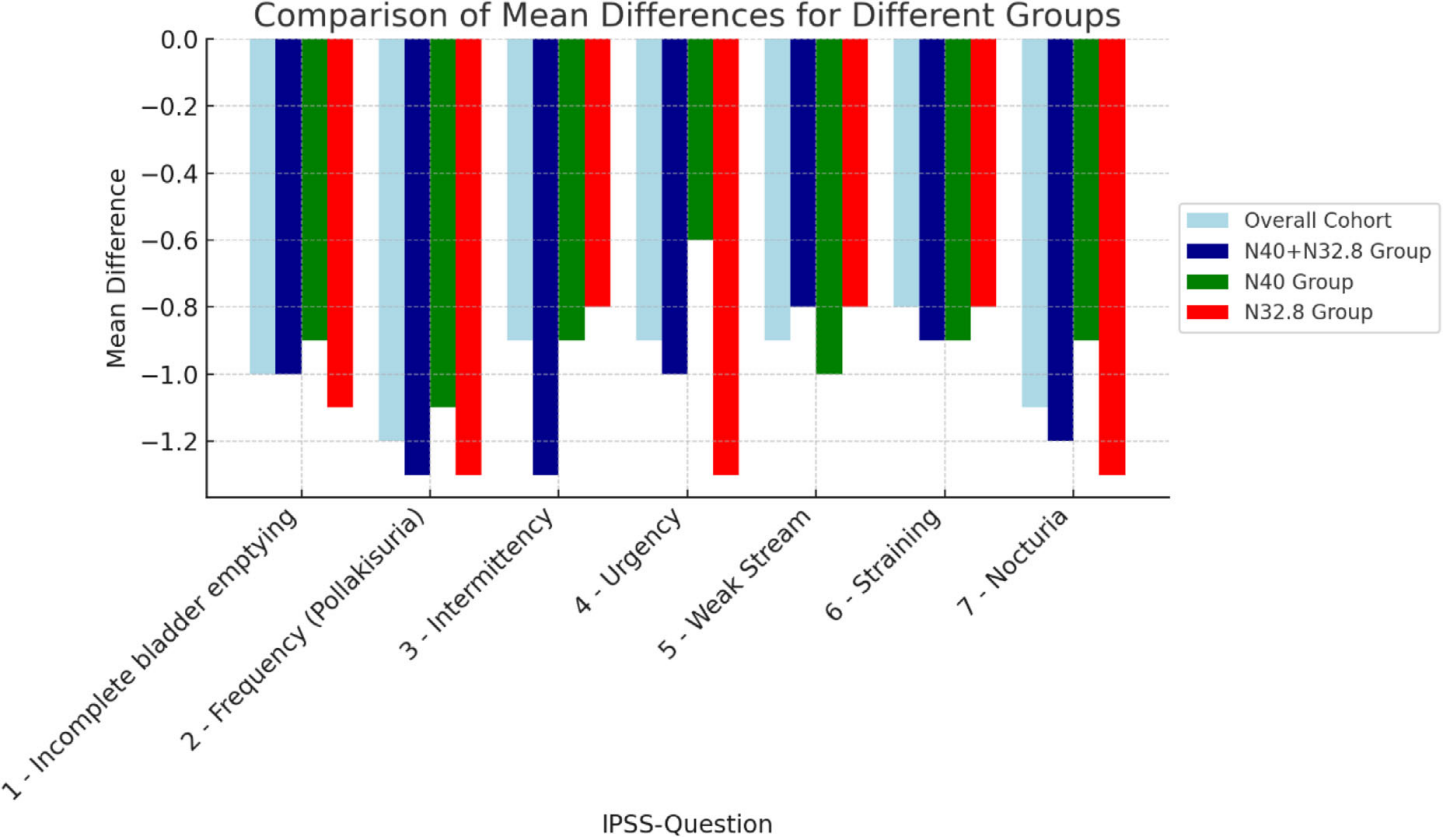
Comparing of the LS Mean Differences for the IPSS single questions for the different groups (overall cohort, BPH + OAB=N40 + N32.8, BPH=N40, OAB=N32.8).

The intervention resulted in a significant improvement in all voiding symptoms according to the IPSS score. The most significant improvements were observed in frequency, nocturia and the feeling of incomplete bladder emptying. Patients with OAB appeared to benefit the most from digital therapy (Figure 1).

## 4 DISCUSSION

Digital health applications, such as Kranus Lutera, have increasingly emerged as valuable adjuncts to therapeutic strategies across various medical disciplines.^2^ These app‐based applications, classified as low‐risk medical devices, serve diagnostic as well as therapeutic purposes by aiding in the identification and alleviation of disease symptoms.^7^ In the context of male LUTS, the app‐based therapy Kranus Lutera has demonstrated efficacy in the randomized controlled BEST‐trial.^3^ This raises the question of which specific voiding symptom domain respond most favourably to the digital interventions. The IPSS questionnaire encompasses both, storage symptoms (questions 2, 4 and 7) and voiding symptoms (questions 1, 3, 5 and 6),^1^ thereby allowing for a nuanced evaluation. Previous studies have also utilized the IPSS for differentiated symptom analysis.^8^, ^9^, ^10^

The general reduction in the IPSS scores observed within the IG suggests that the digital intervention effectively alleviates voiding‐related symptoms. Notably, improvements were most pronounced with regard to urinary frequency, nocturia and the sensation of incomplete bladder emptying, symptoms commonly identified as particularly burdensome by patients.^11^, ^12^, ^13^, ^14^ These storage symptoms are often the primary contributors to the reduced quality of life in individuals with LUTS, highlighting the relevance of the digital therapy's focus on these areas. The LS mean difference values indicate a clinically meaningful benefit, consistent with prior evidence suggesting that digital and behavioural interventions can complement pharmacologic therapies in the management of LUTS. Furthermore, the significant decrease in symptom burden as measured by OAB‐q SF (part 1), alongside concomitant improvements in health‐related quality of life (OAB‐q SF, part 2), underscores the clinical relevance of this digital therapy. Patients reporting fewer urinary symptoms often experience enhancements in overall well‐being, sleep quality and functional capacity in daily life.^14^ These findings support the potential of digital interventions as effective, non‐invasive strategies for improving both symptomatology and quality of life in affected individuals.

A subgroup comparison of patients with overactive bladder (OAB, N32.8) and those with benign prostatic hyperplasia (BPH, N40) revealed that individuals with OAB derived particularly notable benefits from the intervention, especially with respect to urgency and nocturia. This finding suggests heightened efficacy of the digital intervention in addressing storage symptoms. In contrast, patients with isolated BPH, without accompanying storage symptoms, primarily experienced improvements in voiding symptoms, albeit to a lesser extent than those in the OAB group. These differential outcomes emphasize the importance of personalized digital interventions and therapies tailored to the distinct pathophysiological mechanisms underlying various LUTS subtypes.

Previous research has also investigated the utility of mobile health applications in urological contexts, demonstrating their feasibility and clinical potential despite varying therapeutic foci. For example, Goode et al. showed the benefits of a mobile app for pelvic floor training after prostatectomy,^15^ while Wadensten et al. assessed a mobile health tool for behavioural therapy in women with urinary incontinence.^16^ Morselli et al. evaluated the use of a smartphone application for monitoring male LUTS treatment during the COVID‐19 pandemic.^17^ Although these apps targeted different patient populations or clinical scenarios, they collectively support the broader applicability and value of digital tools in urological care.

Therefore, the current study builds upon this growing body of evidence by demonstrating the effectiveness of a certified and structured digital therapy specifically for male LUTS. These findings contribute to the evolving landscape of digital health and support its integration into individualized, symptom‐targeted treatment strategies in urology.

## 5 STRENGTHS AND LIMITATIONS

A key strength of this study lies in its rigorous methodological design, including the use of validated questionnaires (IPSS and OAB‐q SF) and a controlled comparison between an intervention and control group. The multidimensional assessment of symptom domains further enables a comprehensive evaluation of treatment efficacy. Additionally, the inclusion of real‐world data enhances the external validity and applicability of the findings within routine clinical practice, although it also introduces interpretative complexity.

Notably, the study did not incorporate objective urodynamic measures such as uroflowmetry, post‐void residual volume or urodynamic studies. A potential source of selection bias must also be acknowledged, as access to the application required ownership of a smartphone or tablet. Individuals unfamiliar with such technology were thus inherently excluded from participation. Moreover, participants experiencing greater symptom burden may have been more intrinsically motivated to engage in behavioural change, thereby skewing the sample toward more health‐conscious or tech‐savvy individuals. Consequently, generalizability to less motivated, less physically active or less technologically inclined populations may be limited.

In this context, it is important to emphasize that the use of a certified digital therapy app represents a novel, non‐pharmacological treatment approach for LUTS, which inherently requires a certain level of digital literacy and access to compatible devices. This digital prerequisite may contribute to a so‐called digital divide, particularly affecting older populations or individuals with limited technical experience. However, our study data did not reveal any relevant outcome differences with respect to patient age, suggesting that the limiting factor is not the patients' biological age, but rather the availability of a smartphone and the associated cognitive ability to use it. Future implementations and studies should therefore consider barriers to technology adoption and strive for inclusive solutions that address these disparities.

Furthermore, the control group did not employ a sham application, which presents a potential bias, as the use of a generic health app could itself yield beneficial effects. In addition, the study duration of 12 weeks, while adequate to capture meaningful short‐term outcomes, does not permit conclusions regarding the sustainability of observed effects. Future studies incorporating extended follow‐up periods are required to determine the durability of these benefits. In the BEST‐study, 84% of the participants used the app‐based therapy several times a week.

Going forward, research should prioritize evaluating the long‐term effectiveness of digital health applications in managing male LUTS. Comparative studies exploring the combined impact of digital interventions with pharmacotherapy versus pharmacotherapy alone could offer valuable insights into their role in evolving treatment paradigms. Additionally, investigating patient‐specific predictors of favourable responses to digital therapies may facilitate a more individualized and effective approach to care.

## 6 CONCLUSION

This study provides compelling evidence that digital health interventions can significantly reduce LUTS severity, improve symptom burden and enhance quality of life in affected patients. The detailed analysis of IPSS questions highlights that frequency, nocturia and incomplete bladder emptying were the most responsive to the intervention. These symptoms, particularly storage symptoms, are the most bothersome and impactful on quality of life for many patients, underscoring the significance of the intervention's effectiveness in these areas. Additional improvements were observed in interrupted stream, urgency, weak stream and hesitancy. These findings support the integration of digital therapeutic approaches into routine urological care, particularly for patients with OAB symptoms. As digital health continues to evolve, further research is warranted to optimize these interventions and explore their full potential in clinical practice.

## CONFLICT OF INTEREST STATEMENT

Sandra Schönburg and Christian Gratzke have led the BEST (Bladder Emptying DiSorder Therapy)‐study. Kurt Miller is a Medical Advisor of Kranus Health. Mrs. Laura Wiemer is the Senior Medical Director of Kranus Health and Mr. Erik Krieger is the Global Therapeutic Area Lead of Kranus Health.

## References

[1] Cockett AT , Aso Y , Denis L , et al. Empfehlungen des International Consensus Committees. Prog Urol. 1991;1(6):957–972. 1726946

[2] Schönburg S , Gratzke C , Miller K , Wiemer L , Kliesch S . Digitale Gesundheitsanwendungen in der Urologie. Urologe. 2024;63(9):850–859. 10.1007/s00120-024-02398-0 39133296

[3] Gratzke C , Schönburg S , Eger S , Raude K , Grabbert M , Astheimer S , et al. A randomized trial of an app‐based therapeutic for lower urinary tract symptoms. NEJM Evid. 2025;4(4):EVIDoa2400290. 10.1056/EVIDoa2400290 40130971

[4] Coyne K , Revicki D , Hunt T , Corey R , Stewart W , Bentkover J , et al. Psychometric validation of an overactive bladder symptom and health‐related quality of life questionnaire: the OAB‐q. Qual Life Res. 2002;11(6):563–574. 10.1023/a:1016370925601 12206577

[5] Acquadro C , Kopp Z , Coyne KS , Corcos J , Tubaro A , Choo MS , et al. Translating overactive bladder questionnaires in 14 languages. Urology. 2006;67(3):536–540. 10.1016/j.urology.2005.09.035 16527574

[6] Groenendijk IM , Scheepe JR , Noordhoff TC , Blok BFM . The validation of the Dutch OAB‐q SF: An overactive bladder symptom bother and health‐related quality of life short form questionnaire. NeurourolUrodyn. 2019;38(6):1775–1782. 10.1002/nau.24074 31215693

[7] Ludewig G , Klose C , Hunze L , Matenaar S . Digital health applications: Statutory introduction of patient‐centred digital innovations into healthcare. Bundesgesundheitsblatt. 2021;64(10):1198–1206. 10.1007/s00103-021-03407-9 PMC8492586 34529096

[8] Lyauk YK , Jonker DM , Lund TM , Hooker AC , Karlsson MO . Item response theory modeling of the International Prostate Symptom Score in patients with lower urinary tract symptoms associated with benign prostatic hyperplasia. AAPS J. 2020;22(5):115. 10.1208/s12248-020-00500-w 32856168 PMC7452927

[9] Yokoyama O , Ozeki A , Suzuki N , Murakami M . Early improvement of storage or voiding symptoms by tadalafil predicts treatment outcomes in patients with lower urinary tract symptoms from benign prostatic hyperplasia. Int J Urol. 2018;25(3):240–245. 10.1111/iju.13487 29094398

[10] D'Agate, S , Development of a drug‐disease model describing individual IPSS trajectories in BPH patients: implication of disease progression and covariate factors on long term treatment response. PAGE 2018; III‐77.

[11] Homma Y , Yamaguchi T , Kondo Y , Horie S , Takahashi S , Kitamura T . Significance of nocturia in the International Prostate Symptom Score for benign prostatic hyperplasia. J Urol. 2002;167(1):172–176. 10.1016/S0022-5347(05)65406-7 11743299

[12] Everaert K , Anderson P , Wood R , Andersson FL , Holm‐Larsen T . Nocturia is more bothersome than daytime LUTS: Results from an observational, real‐life practice database including 8659 European and American LUTS patients. Int J Clin Pract. 2018;72(6):e13091. 10.1111/ijcp.13091 29767479

[13] Lee JY , Lee DH , Lee H , Bang WJ , Hah YS , Cho KS . Clinical implications of a feeling of incomplete emptying with little post void residue in men with lower urinary tract symptoms. NeurourolUrodyn. 2014;33(7):1123–1127. 10.1002/nau.22473 23946081

[14] Agarwal A , Eryuzlu LN , Cartwright R , Thorlund K , Tammela TL , Guyatt GH , et al. What is the most bothersome lower urinary tract symptom? Individual‐ and population‐level perspectives for both men and women. Eur Urol. 2014;65(6):1211–1217. 10.1016/j.eururo.2014.01.019 24486308 PMC4018666

[15] Goode P , Johnson TM , Newman DK , Goode PS , Vaughan CP , Echt KV , et al. Perioperative mobile telehealth program for post‐prostatectomy incontinence: A randomized clinical trial. J Urol. 2022;208(2):379–387. 10.1097/JU.0000000000002697 35389239

[16] Wadensten T , Nyström E , Franzén K , Lindam A , Wasteson E , Samuelsson E . A mobile app for self‐management of urgency and mixed urinary incontinence in women: Randomized controlled trial. J Med Internet Res. 2021;23(4):e19439. 10.2196/19439 33818395 PMC8056293

[17] Morselli S , Liaci A , Nicoletti R , Pecoraro A , Gemma L , Polverino P , et al. The use of a novel smartphone app for monitoring male LUTS treatment during the COVID‐19 outbreak. Prostate Cancer Prostatic Dis. 2020;23(4):724–726. 10.1038/s41391-020-0253-z 32665609 PMC7359441

